# High Prevalence of β-lactamase and Plasmid-Mediated Quinolone Resistance Genes in Extended-Spectrum Cephalosporin-Resistant *Escherichia coli* from Dogs in Shaanxi, China

**DOI:** 10.3389/fmicb.2016.01843

**Published:** 2016-11-16

**Authors:** Xiaoqiang Liu, Haixia Liu, Yinqian Li, Caiju Hao

**Affiliations:** ^1^Department of Basic Veterinary Medicine, College of Veterinary Medicine, Northwest A&F UniversityYangling, China; ^2^Department of Aquaculture, College of Animal Science and Technology, Northwest A&F UniversityYangling, China

**Keywords:** *Eshcerichia coli*, β-lactamases, PMQR, cephalosporin resistance, dog

## Abstract

**Objective:** The aim of this study was to investigate the occurrence and molecular characterization of extended-spectrum β-lactamases (ESBL), plasmid-mediated AmpC β-lactamase (pAmpC) and carbapenemases as well as plasmid-mediated quinolone-resistant (PMQR) among extended-spectrum cephalosporin-resistant (ESC-R) *Escherichia coli* from dogs in Shaanxi province in China.

**Methods:** A total of 40 ESC-R *Escherichia coli* selected from 165 Extraintestinal pathogenic *E. coli* (ExPEC) isolated from dogs were screened and characterized for the genes encoding for the ESBLs, pAmpC, carbapenemases and PMQR genes by PCR and sequencing. Phylogenetic groups, virulence gene profiles and multilocus sequence typing (MLST) were used to investigate the genetic background of the ESC-R *E. coli* isolates.

**Results:** Among 40 ESC-R *E. coli*, the predominant β-lactamase gene was *bla*_CTX−Ms_ (*n* = 35), and followed by *bla*_TEM−1_ (*n* = 31), *bla*_SHV−12_ (*n* = 14), *bla*_OXA−48_ (*n* = 8), *bla*_TEM−30_ (*n* = 4), *bla*_CMY−2_ (*n* = 3) and *bla*_DHA−1_ (*n* = 2). The most common specific *bla*_CTX−M_ gene subtype was *bla*_*CTX*−*M*−15_ (*n* = 31), and followed by *bla*_CTX−M−123_ (*n* = 14), *bla*_CTX−M−1_ (*n* = 10), *bla*_CTX−M−14_ (*n* = 10) and *bla*_CTX−M−9_ (*n* = 7). PMQR genes were detected in 32 (80%) isolates, and the predominant PMQR gene was *aac(6*′*)-Ib-cr* (*n* = 26), followed by *qnrS* (*n* = 12), *qnrD* (*n* = 9), *qnrB* (*n* = 8), *qepA* (*n* = 4), and all PMQR genes were detected in co-existence with β-lactamase genes. *traT* (*n* = 34) and *fimH* (*n* = 32) were the most prevalent virulence genes, and virulence genes *fimH, iutA, fyuA, malX, iha*, and *sat* were more prevalent in phylogenetic group B2. The 40 ESC-R isolates analyzed were assigned to 22 sequence types (STs), and the clonal lineages ST131 (*n* = 10) and ST10 (*n* = 9) were the predominant STs.

**Conclusion:** High prevalence of β-lantamases and PMQR genes were detected among ESC-R *E. coli* from companion animals. This is also the first description of the co-existence of six β-lantamase genes and five PMQR genes in one *E. coli* isolate. Moreover, 10 ST131 clones harboring CTX-M-15 were detected.

## Introduction

*Escherichia coli* is one of the most predominant organisms causing infections in human and animals. The β-lactams, particularly the third-generation cephalosporins are important for the treatment of serious infections in companion animals caused by *Enterobacteriaceae* (Jiang et al., 2012). However, there has been an increasing number of infections worldwide due to the third-generation cephalosporin resistant *E. coli* isolates worldwide, and companion animals can service as a reservoir of cephalosporin-resistant bacteria as the physical closeness between humans and their pet companions (Costa et al., 2009; Wasyl et al., 2012). One of the currently most important resistance mechanisms in *E. coli* is based on plasmid mediated production of enzymes, especially the extended-spectrum β-lactamases (ESBL), which inactivate β-lactams by hydrolyzing their β-lactam rings (Zhao and Hu, 2013). Additionally, plasmid-mediated AmpC β-lactamase (pAmpC) *bla*_CMY−2_, carbapenemases *bla*_NDM−1_, *bla*_OXA−48_ and *bla*_KPC−2_ are now also increasingly described (Nordmann et al., 2011). β-lactamase genes are usually located on mobile genetic elements, such as plasmids, transposons or integrons. Resistant plasmids are transferred between bacterial isolates or different bacterial species by conjugation mechanism (Cantas et al., 2015). Furthermore, extended-spectrum cephalosporin-resistant (ESC-R) *E. coli* isolates are often cross-resistant to fluoroquinolones, sulfamethoxazole/trimethoprim or other antimicrobials, and finally expressed multidrug resistance (MDR) (Tian et al., 2009, 2012).

The emergence of plasmid-mediated quinolone resistance (PMQR) indicated that quinolone resistance can also be acquired through horizontal gene transfer, and PMQR genes can co-exist on the same plasmid with genes encoding ESBLs and to be co-transferred to recipients (Briales et al., 2012; Yu et al., 2015). Moreover, PMQR genes can create an environment in *E. coli* for the rapid selection of high level resistance, although they usually just confer the lower-level resistance (Ishida et al., 2010).

Although, the ESBLs and other β-lactamases in *E. coli* isolates from humans and food-animals have been characterized in various studies in China (Yuan et al., 2009; Sun et al., 2010; Wang et al., 2012; Xi et al., 2015; Xu et al., 2015; Yu et al., 2015), very little data has been reported on the occurrence of β-lactamases and PMQR as well as the population structure in ExPEC isolates from companion animals in Shaanxi province, even in China. This prompted us to determine and characterize the β-lactamases (ESBLs, pAmpC, and carbapenemases) and PMQR genes in ESC-R *E. coli* collected from dogs in Shaanxi province, and shed light on the phylogenetic groups, virulence gene profiles and the multilocus sequence typing (MLST).

## Materials and methods

### Bacterial isolates

A collection of 165 unique ExPEC isolates were isolated from urine, wound, genital tract, anal sac, nasal structure and soft tissue samples of dogs with naturally-occurring infection in the small animal hospitals in Shaanxi province, China from March 2013 to October 2015. Samples were collected from individual dog using a sterile swab and transported to the laboratory within 12 h. Samples were immediately seeded on MacConkey agar at 37°C, and one colony with typical *E. coli* morphology was selected from each sample. *E. coli* were identified using API-20E (BioMerieux, Beijing, China). All confirmed *E. coli* isolates were stored at −80°C in the Tryptic Soy broth medium containing 30% glycerol for further study.

### Antimicrobial susceptibility testing

Antimicrobial susceptibility testing was performed for all 165 isolates. The minimum inhibitory concentrations (MICs) of third-generation cephalosporins (ceftiofur, cefotaxime, cefoperazone, cefpodoxime, ceftazidime), fluoroquinolones (enrofloxacin, ciprofloxacin, and pradofloxacin) and other β-lactams (ampicillin, amoxicillin-clavulanic acid, cefoxitin, doxycycline, imipenem, meropenem, sulfamethoxazole/trimethoprim, gentamicin, and amikacin) were determined by a standardized microdilution method followed the CLSI guidelines (CLSI, 2013). All MIC determinations were performed in triplicates and *E. coli* ATCC 25922 was used for quality control.

Additionally, all the 165 *E. coli* isolates were screened for ESBL production using microdilution-based Sensititre (TREK diagnostic systems, Cleveland, Ohio) with ESBL Confirmatory MIC plates (ESB1F) as described previously (Aly et al., 2012).

### Phylogenetic typing and virulence genotyping

Genomic DNA were extracted from bacterial preparations using the PreMan® Ultra Preparation Reagent according to manufacturer's protocol. The pathogenicity of *E. coli* is associated with the presence of virulence genes that can be encoded by chromosomal or plasmid genes, and thus each isolate was examined for the presence of 19 virulence genes (Table S1) known for their association with ExPEC by use of established PCR assays. This panel of genes represent five categories: adhesins genes including *fimH, sfa/focDE, afa/draBC, iha, papA, papC, papG*, and *papG alleles* (I, II, III); toxin genes including *hlyA, cnf1* and *sat*; capsule gene including *kpsM* II; siderophore genes includinge *fyuA* and *iutA*; the miscellaneous virulence genes including *traT* and *malX*.

### Identification of β-lactamase genes and plasmid-mediated quinolone resistance genes

The occurrence of ESBLs (TEM, SHV, CTX-Ms), pAmpC (CMY-2, DHA-1, FOX, ACC, and EBC) and carbapenemase genes (class B, NDM-1; class A, KPC-2; class D, OXA-48) in ESC-R *E. coli* were determined by PCR using specific primers (Table S1) and conditions previously described (Perez-Perez and Hanson, 2002; Yan et al., 2004; Poirel et al., 2011; Shaheen et al., 2011). Meanwhile, all ESC-R *E. coli* isolates were characterized by PCR for PMQR genes (*qnrA, qnrB, qnrD, qnrS, aac(6*′*)-Ib-cr*, and *qepA*) as described previously (Liu et al., 2012). All PCR products from ESBL and PMQR genes were confirmed and analyzed by DNA sequencing.

### Multilocus sequence typing (MLST)

MLST was performed using seven conserved housekeeping genes of *E. coli* (*adk, fumC, gyrB, icd, mdh, purA*, and *recA*). A detailed scheme of the MLST procedure is available at MLST databases at the at the Warwick University website (http://mlst.warwick.ac.uk/mlst/dbs/Ecoli).

### Pulsed-field gel electrophoresis (PFGE) analysis

In order to determine the genetic relatedness of the ESC-R *E. coli*, the chromosomal DNA of some selected isolates were digested with the restriction enzyme *Xba*I and then subjected to PFGE analysis according to the PulseNet protocol of the US Centers for Disease Control and Prevention (http://www.cdc.gov/pulsenet/protocols.htm) and previous literature (Tenover et al., 1995). The strain of *Salmonella* serotype Braenderup restricted with *Xba*I was used as molecular weight standard.

### Conjugation experiments

The transferability of β-lactamase and PMQR genes was performed by mating on 10 randomly selected ESC-R *E. coli* isolates with azide-resistant *E. coli* J53 (J53 AZ^r^) as described previously (Loncaric et al., 2013). Transconjugants were selected on Mueller-Hinton (MH) agar supplemented with 150 μg/ml sodium azide and 2 μg/ml cefotaxime. Antimicrobial susceptibility, confirmatory tests for ESBL production, and PCR detection were performed on all transconjugants to confirm transfer of relevant β-lactamase and PMQR genes.

## Results

### Antimicrobial susceptibility of *E. coli* isolates

Among the 165 *E. coli* isolates surveyed, 40 (24.2%) isolates were both ESC-R and ESBL-producing isolates, which exhibited high resistance rate to cefotaxime (100%), ceftazidime (87.5%), ceftoxitin (62.5%), cefoperazone (52.5%), and also exhibited high resistance rate to amoxicillin-clavulanic acid (90%). The frequencies of resistance to non-β-lactam antibiotics were as follows: doxycycline, 95%; enrofloxacin, 92.5%; sulfamethoxazole/trimethoprim, 77.5%; chloramphenicol, 70%; gentamicin, 60%; pradofloxacin, 55%. Additionally, it was noteworthy that 10 (25%) isolates were resistance to imipenem and meropenem, respectively. The resistance rates to other antibacterial agents tested were lower than 40%, and all ESC-R *E. coli* isolates exhibited the MDR phenotype

### Phylogenetic typing and virulence genotyping

Phylogenetic group analysis revealed that the predominant phylogenetic group was A (37.5%) and B2 (35%), followed by phylogenetic groups D (12.5%), E (7.5%), B1 (5%), and F (2.5%) among 40 ESC-R isolates tested (Table 1). Eighteen of 19 investigated virulence genes were detected, and the overall prevalence of the virulence genes ranged from 7.5% (*papG*) to 85% (*traT*). *fimH, iutA, fyuA*, and *malX* were more prevalent in phylogenetic group B2, but less prevalent in group A isolates (*P* < 0.001), and *iha* and *sat* exclusively present in group B2 (Table 1).

**Table 1:** **The occurrence of virulence genes in ESC-R ***E. coli*** isolates**.

**Isolate**	**PG**	***fimH***	***papA***	***papC***	***papG***	***papG*III**	***afa/draBC***	***Sfa/focDE***	***hlyA***	***cnf1***	***iutA***	***fyuA***	***traT***	***ibeA***	***malX***	***sat***	***iha***	***iroN***	***kpsM* II**
B1	A			+									+						
H2	A			+						+			+						
W12	A	+											+						
B6	A			+						+		+	+						
Y5	A	+		+							+		+					+	
W4	A	+		+									+						
X2	A	+		+							+		+					+	
Y15	A	+			+	+	+	+			+	+		+				+	
B3	A										+	+	+		+				
W15	A			+									+						
W6	A	+										+	+						
Y13	A	+											+					+	
X1	A			+									+						
B5	A							+	+		+		+		+				+
H1	A												+						
X5	B1	+											+					+	
X6	B1	+		+									+	+					
Y10	B2	+		+									+						
X8	B2	+	+	+					+		+	+	+		+	+	+	+	+
W19	B2	+	+	+								+	+		+	+	+		
W9	B2	+									+	+	+		+	+	+	+	+
Y8	B2	+	+	+		+	+				+	+	+		+				
X9	B2	+		+			+	+			+	+	+	+	+				
X16	B2	+									+		+		+				
Y11	B2	+		+	+		+	+			+	+	+		+	+	+		
Y17	B2	+		+								+	+				+		
W13	B2	+	+	+								+	+		+	+	+	+	
Y4	B2	+	+	+				+		+	+	+			+				
X7	B2	+	+	+	+	+					+	+	+	+	+	+	+	+	
W11	B2	+	+	+		+			+		+	+	+		+	+	+		
H6	B2	+					+					+	+		+	+	+		
X12	D	+	+	+								+	+		+				
X15	D	+	+	+								+	+		+				
X10	D	+				+		+		+		+						+	
X18	D	+	+	+		+					+	+	+		+				
X27	D	+	+	+					+			+	+				+	+	+
Y7	E	+		+															
W8	E	+										+							
W13	E	+	+								+	+	+		+			+	
H5	F	+										+						+	

*papGI and papGII were not detected*.

### Characterization of β-lactamases and PMQR determinants

All ESC-R *E. coli* tested harbored one or more β-lactamase genes. *bla*_TEM_, *bla*_SHV_, *bla*_CTX−M_, *bla*_CMY−2,_ and *bla*_DHA−1_ were determined in 35 (87.5%), 14 (35%), 35 (87.5%), 3 (7.5%), and 2 (5%) of ESC-R *E. coli* isolates, respectively (Table 2). Moreover, eight *bla*_OXA−48_ genes associated with carbapenems (imipenem and meropenem) resistance were detected. For the *bla*_CTX−M_ positive isolates, *bla*_CTX−M−15_ (*n* = 35) was the predominant specific subtype, and followed by *bla*_CTX−M−123_ (*n* = 14), *bla*_CTX−M−1_ (*n* = 10), *bla*_CTX−M−14_ (*n* = 10), and *bla*_CTX−M−9_ (*n* = 7). Sequencing of *bla*_TEM_ gene revealed 31 *bla*_TEM−1_ and four *bla*_TEM−30_, whereas sequencing of *bla*_SHV_ gene revealed 14 *bla*_SHV−12_. Eighty percent (32/40) of tested isolates harbored at least one PMQR gene, and 16 isolates harbored more than one PMQR genes. *qnrB, qnrD, qnrS, qepA*, and *aac(6*′*)-Ib-cr* were detected alone or incombination in 20, 22.5, 30, 10, and 65% of 40 ESC-R isolates, respectively, while *qnrA* was not detected (Table 2). Furthermore, all PMQR genes were detected in co-existence with β-lactamase genes, and one isolate harbored simultaneously β-lactamase genes *bla*_TEM−1_, *bla*_SHV−12_, *bla*_CTX−M−15_, *bla*_CTX−M−9_, *bla*_CTX−M−14_, *bla*_DHA−1,_ and *bla*_OXA−48_ as well as PMQR genes *qnrB, qnrD, qnrS, qepA*, and *aac(6*′*)-Ib-cr*.

**Table 2:** **The occurrence of β-lactamases and PMQR genes in ESC-R ***E. coli*** isolates**.

**Isolates ID**	**PG**	**MLST**	**β-lactamase genes**				**PMQR genes**	**Resistance profiles**
			**Non-ESBL**	**ESBL**	**pAmpC**	**Carbapenemase**		
B1	A	ST1820		TEM-30			*qnrS, aac(6′)-Ib-cr*	CFZ, CTX, CPO, ENR, DOX, GEN
H2	A	ST746	TEM-1	CTX-M-14				AMC, CFZ, CTX, CAZ, ENR, DOX, SXT
W12	A	ST44	TEM-1	CTX-M-15				AMC, CFZ, CTX, CAZ, ENR, DOX, CHL
B6	A	ST1700	TEM-1	CTX-M-1, CTX-M-15, CTX-M-123			*aac(6′)-Ib-cr*	AMC, CFZ, CTX, CAZ, CPD, CRO, FOX, ENR, PRA, DOX, AMK, SXT
Y5	A	ST167	TEM-1	SHV-12			*qnrD, qnrS*	AMC, CTX, ENR, DOX, CHL, GEN
W4	A	ST167	TEM-1	CTX-M-9, CTX-M-14, CTX-M-123		OXA-48	*qnrD, qnrS*	AMC, CFZ, CTX, CAZ, CPD, CRO, CPO, FOX, MEM, ENR, PRA, DOX, CHL, GEN, AMK, SXT
X2	A	ST10	TEM-1	CTX-M-15			*qnrD, qnrS*	AMC, CFZ, CTX, CAZ, ENR, DOX, SXT
Y15	A	ST10		SHV-12, CTX-M-15			*qnrB, qnrD*	AMC, CTX, CAZ, CPD, CPO, FOX, ENR, DOX, CHL, GEN, SXT
B3	A	ST10		SHV-12, CTX-M-15			*qnrB, aac(6′)-Ib-cr*	AMC, CTX, CAZ, CPD, CRO, CPO, ENR, PRA, DOX, GEN, AMK, SXT
W15	A	ST10	TEM-1	SHV-12, CTX-M-15		OXA-48		CFZ, CTX, CAZ, CPD, CRO, FOX, IPM, DOX, CHL, GEN, SXT
W6	A	ST10		SHV-12, CTX-M-15, CTX-M-14			*qnrB, qepA, aac(6′)-Ib-cr*	AMC, CFZ, CTX, CAZ, CRO, CPO, FOX, ENR, PRA, DOX, CHL, SXT
Y13	A	ST10	TEM-1	CTX-M-15, CTX-M-9, CTX-M-14			*qnrB, qnrD, qnrS, aac(6′)-Ib-cr*	AMC, CFZ, CTX, CAZ, CPD, CRO, CPO, FOX, ENR, PRA, DOX, CHL, GEN, AMK, SXT
X1	A	ST10	TEM-1	CTX-M-1, CTX-M-15, CTX-M-123			*qnrS, aac(6′)-Ib-cr*	AMC, CFZ, CTX, CAZ, CPD, CRO, CPO, FOX, ENR, PRA, DOX, CHL, SXT
B5	A	ST10	TEM-1	CTX-M-1, CTX-M-15, CTX-M-123			*qnrD, qnrS, aac(6′)-Ib-cr*	AMC, CFZ, CTX, CAZ, CPD, CRO, CPO, FOX, ENR, PRA, DOX, CHL, SXT
H1	A	ST10	TEM-1	CTX-M-1, CTX-M-15, CTX-M-123	CMY-2		*qnrS, aac(6′)-Ib-cr*	AMC, CTX, CAZ, CRO, CPO, FOX, IPM, ENR, PRA, DOX, CHL, GEN, AMK, SXT
X5	B1	ST75		SHV-12, CTX-M-15			*aac(6′)-Ib-cr*	AMC, CTX, CAZ, CRO, ENR, CHL, SXT
X6	B1	ST1177	TEM-1	CTX-M-15, CTX-M-123				AMC, CFZ, CTX, CAZ, CPD, CRO, CPO, DOX, CHL, SXT
Y10	B2	ST375		TEM-30			*aac(6′)-Ib-cr*	AMC, CTX, CAZ, ENR
X8	B2	ST302	TEM-1	CTX-M-15			*aac(6′)-Ib-cr*	AMC, CFZ, CTX, CAZ, ENR, DOX, GEN
W19	B2	ST73	TEM-1	CTX-M-1, CTX-M-15, CTX-M-123			*aac(6′)-Ib-cr*	AMC, CFZ, CTX, CAZ, CPD, CRO, FOX, ENR, DOX, CHL
W9	B2	ST104	TEM-1	CTX-M-1, CTX-M-9, CTX-M-14		OXA-48		AMC, CFZ, CTX, CAZ, CPD, CRO, CPO, FOX, MEM, ENR, DOX, CHL, GEN, SXT
Y8	B2	ST131	TEM-1	CTX-M-15			*aac(6′)-Ib-cr*	AMC, CTX, CAZ, CRO, ENR, PRA, DOX
X9	B2	ST131	TEM-1	CTX-M-15	DHA-1		*qnrD, qnrS*	AMC, CFZ, CTX, CAZ, CPD, CRO, CPO, FOX, MEM, ENR, PRA, DOX, CHL, SXT
X16	B2	ST131	TEM-1	CTX-M-15		OXA-48	*qnrS, qepA, aac(6′)-Ib-cr*	AMC, CFZ, CTX, CAZ, CPD, CRO, CPO, FOX, IPM, ENR, PRA, DOX, CHL, GEN, AMK, SXT
Y11	B2	ST131	TEM-1	SHV-12, CTX-M-15			*qnrB*	AMC, CFZ, CTX, ENR, DOX, SXT
Y17	B2	ST131	TEM-1	SHV-12, CTX-M-15			*aac(6′)-Ib-cr*	AMC, CFZ, CTX, CAZ, CPO, FOX, ENR, PRA, DOX, SXT
W13	B2	ST131	TEM-1	CTX-M-1, CTX-M-15			*aac(6′)-Ib-cr*	AMC, CFZ, CTX, CAZ, CPD, CRO, FOX, ENR, DOX, SXT
Y4	B2	ST131	TEM-1	CTX-M-15, CTX-M-123			*qnrS, aac(6′)-Ib-cr*	CFZ, CTX, CAZ, CPD, CRO, CPO, FOX, ENR, PRA, DOX, CHL, GEN, AMK, SXT
X7	B2	ST131	TEM-1	SHV-12, CTX-M-15, CTX-M-123	CMY-2		*qnrB, qnrD, aac(6′)-Ib-cr*	AMC, CFZ, CTX, CAZ, CPD, CRO, CPO, FOX, ENR, PRA, DOX, CHL, GEN, AMK, SXT
W11	B2	ST131	TEM-1	SHV-12, CTX-M-1, CTX-M-15, CTX-M-123	CMY-2		*aac(6′)-Ib-cr*	AMC, CFZ, CTX, CAZ, CPD, CRO, CPO, FOX, ENR, PRA, DOX, CHL, GEN, AMK, SXT
H6	B2	ST131	TEM-1	CTX-M-1, CTX-M-15, CTX-M-9, CTX-M-14, CTX-M-123			*aac(6′)-Ib-cr*	AMC, CFZ, CTX, CAZ, CPD, CRO, FOX, ENR, PRA, DOX, CHL, GEN, AMK, SXT
X12	D	ST69	TEM-1	CTX-M-15, CTX-M-123		OXA-48	*aac(6′)-Ib-cr*	AMC, CFZ, CTX, CAZ, CPD, CRO, CPO, FOX, MEM, ENR, PRA, DOX, CHL, GEN, AMK, SXT
X15	D	ST38	TEM-1	CTX-M-15, CTX-M-9, CTX-M-14				AMC, CFZ, CTX, CAZ, CPD, CRO, DOX, CHL, SXT
X10	D	ST405	TEM-1	SHV-12, CTX-M-15, CTX-M-14, CTX-M-123		OXA-48	*aac(6′)-Ib-cr*	AMC, CFZ, CTX, CAZ, CPD, CRO, CPO, FOX, IPM, ENR, PRA, DOX, CHL, GEN, AMK, SXT
X18	D	ST648	TEM-1	SHV-12, CTX-M-15, CTX-M-9, CTX-M-14	DHA-1	OXA-48	*qnrB, qnrD, qnrS*, qepA, aac*(6′)-Ib-cr*	AMC, CFZ, CTX, CAZ, CPD, CRO, CPO, FOX, IPM, ENR, PRA, DOX, CHL, GEN, AMK, SXT
X27	D	ST68		TEM-30				CTX, ENR, DOX
Y7	E	ST1421		TEM-30			*qnrB, aac(6′)-Ib-cr*	AMC, CFZ, CTX, CPD, ENR, PRA, DOX
W8	E	ST2375		SHV-12, CTX-M-15				AMC, CFZ, CTX, CAZ, CPD, DOX, CHL, SXT
W13	E	ST3058	TEM-1	SHV-12, CTX-M-15		OXA-48	*aac(6′)-Ib-cr*	AMC, CFZ, CTX, CAZ, CPD, CRO, CPO, FOX, MEM, ENR, DOX, CHL, GEN, AMK, SXT
H5	F	ST3630	TEM-1	CTX-M-1, CTX-M-9, CTX-M-14, CTX-M-123			*qepA, aac(6′)-Ib-cr*	AMC, CFZ, CTX, CAZ, CPD, CRO, FOX, ENR, PRA, DOX, CHL, SXT

*AMC, amoxicillin-clavulanic acid; CFZ, cefazolin; CTX, cefotaxime; CAZ, ceftazidime; CPD, cefpodoxime; CRO, ceftriaxone; CPO, cefoperazone; FOX, cefoxitin; IPM, imipenem; MEM, meropenem; ENR, enrofloxacin; PRA, pradofloxacin; DOX, doxycycline; CHL, chloramphenicol; GEN, gentamicin; AMK, amikacin; SXT, sulfamethoxazole-trimethoprim*.

### MLST profiles

The 40 ESC-R *E. coli* isolates tested were assigned to 22 STs (Table 2), and ST131 (*n* = 10) of phylogenetic group B2 and ST10 (*n* = 9) of phylogenetic group A accounted for almost 50% of isolates. Two isolates were assigned to ST167. The remaining isolates exhibited diverse ST types. All ST131 and ST10 isolates expressed resistance to ESC-R *E. coli* tested, and they were also ST10 positively associated with *bla*_CTX−M−15_. Furthermore, the ST131 and ST10 isolates were analyzed by PFGE in order to determine the genetic relatedness, PFGE indicated that some ST131 and ST10 isolates displayed the same PFGE profile (Figure S1).

### Conjugation experiments

Five transconjugants were obtained finally, the parental isolates successfully transferred the *bla* genes to the *E. coli* J53 AZ^r^ recipient strain. Moreover, *qnrS* and *aac-(6*′*)-Ib-cr* were co-transferred with *bla*_CTX−M_, whereas other PMQR genes were not observed among transconjugants. The transconjugants had resistance profiles similar to those of their parental isolates. All transconjugants showed high-level resistance to β-lactam antibiotics at the same level as the donor strain. For fluoroquinolones, the transconjugants showed 4–8-fold increases in the MICs of ciprofloxacin when compared with the donor strain *E. coli* J53 AZ^r^. However, the transconjugants remained susceptible to gentamicin, doxycycline and trimethoprim-sulfamethoxazole.

## Discussion

Cephalosporin-resistant *E. coli* isolates seem to be the emergent cause of serious infections of humans and animals worldwide. The present study firstly demonstrated the widespread occurrence and molecular characterization of β-lactamases and PMQR genes among ESC-R *E. coli* isolates from dogs in Shaanxi province, China. The previous studies showed that the occurrence of ESBL carriage in companion animals varies considerably between countries from 1 to 55% (O'keefe et al., 2010; Hordijk et al., 2013), and our data showed that 24.2% of isolates from dogs were ESC-R *E. coli*, which is close to the prevalence (22.3%) reported in the retail meat in Shaanxi province (Xi et al., 2015). While it is significantly higher than the prevalence in the United States (3%; Shaheen et al., 2011) and Switzerland (7.5%; Huber et al., 2013; *P* < 0.0001), and much lower than that in The Netherlands (45–55%) and Guangdong province of China (54.5%) (Sun et al., 2010; Hordijk et al., 2013). The possible reason for this obvious difference is that the difference geographical origin of the isolates, the differences in study population and associated antimicrobial selective pressure. The fluoroquinolones and the third-generation cephalosporins are important for the treatment of serious infections in humans and companion animals caused by Enterobacteriaceae (Jiang et al., 2012), while ESBL-producing *E. coli* isolates associated with MDR phenotype continuously increased with the wide use of cephalosporins, fluoroquinolones and other antimicrobials.

All isolates in the current study expressed MDR phenotype, and exhibited high resistance rates to cefotaxime (100%) and enrofloxacin (95%). Furthermore, it was noteworthy that 10 ESC-R *E. coli* isolates were resistance to meropenem and imipenem agreeing with a previous study in the United States (Liu et al., 2016). It is worrisome although they were still the most potent and effective antibiotics in our study. Owing to the relevance of imipenem and meropenem in the treatment of infections caused by ESBL-positive or MDR *E. coli*, the surveillance of carbapenems resistance and the implementation of guidelines for the rational use of carbapenems will be urgently needed to prevent the progress of antimicrobial resistance in China. Our results showed that ESC-R *E. coli* isolates from dogs belonged mainly to phylogroup A and B2, and to a lesser extent, to phylogenetic D, whereas groups B1, F, and E isolates were very little. The previous studies showed that *E. coli* from pigs or duck in China also mainly felled into phylogenetic groups A (Wang et al., 2010; Ma et al., 2012). While another study has reported that the *E. coli* from cats in the United States belonged predominantly to phyogenetic group B2 (Liu et al., 2015). In regards to the linkage of phylogenetic group and the PMQR genes and β-lactamases, isolates of group A harbored more PMQR genes, and the isolates of group B2 harbored more β-lactamases.

The *bla*_CTX−M_ was the dominating ESBL gene in the *E. coli* tested despite the specific genotype of *bla*_*CTX*−*M*_ are undergoing great changes. *bla*_CTX−M_ was detected in 87.5% of ESC-R *E. coli* isolates, and *bla*_CTX−M−15_ was the most common specific CTX-M subtype in the present study. While *bla*_CTX−M−14_ subtype was the most common CTX-M enzyme in the *E. coli* from food animals in China according to the previous study (Yu et al., 2015). It was worth noting that *bla*_OXA−48_ genes were detected in eight of the 40 (20%) ESC-R *E. coli* isolates, and they were also associated with imipenem or meropenem resistance as a previous study described (Liu et al., 2016). *bla*_OXA−48_ was firstly discovered in *E. coli* from dogs in Germany in 2013, and then it was reported in *E. coli* from companion animals in the United States in 2016 (Stolle et al., 2013; Liu et al., 2016). To our knowledge, this is the first report of *bla*_OXA−48_ in *E. coli* from dogs in China. Although carbapenemase is not an ESBL, *bla*_OXA−48_ has emerged as a major carbapenemase associated with the *Enterobacteriaceae*, and it can also hydrolyze carbapenems and hydrolyzes β-lactamase inhibitors (Mathers et al., 2013). Emergence of PMQR genes have been reported worldwide and is being documented in ESBL-producing *E. coli* (Xu et al., 2015). In the present study, PMQR genes were present in 80% of ESC-R *E. coli* isolates, and *aac(6*′*)-Ib-cr* was detected alone or in combination with *qnr* and *qepA* in present in 65% of isolates, and it occurred in ESBL producers with a higher prevalence than *qnr* genes, especially in *E. coli* isolates carrying the *bla*_CTX−M−15_ gene. It was consistent with a previous study that *aac(6*′*)-Ib-cr* gene was the predominant PMQR gene among *Enterobacteriaceae* isolates from companion animals in Guangdong province in China (Ma et al., 2009). While *oqxAB* was the predominant PMQR gene in *E. coli* from food animals in China (Liu et al., 2013; Xu et al., 2015). In addition, *qepA* gene was detected in combination with other PMQR genes and ESBL genes in four isolates (10%). The presence of PMQRs is of great importance because they are not merely able to confer resistance against fluoroquinolones but as well are often related to ESBLs and/or AmpC β-lactamases. Emergence of PMQR gene *qnr, aac(6*′*)-Ib-cr* and *qepA* has been reported worldwide and were often found to be strongly associated with ESBL genes even located on the same plasmid (Strahilevitz et al., 2009; Poirel et al., 2012). Our results showed that *qnrS* and *aac-(6*′*)-Ib-cr* were co-transferred with *bla*_CTX−M_ in the conjugation experiments, and more studies should be carried out in the future in order to ensure that the six PMQR genes were located on the same plasmid or not. However, we cannot find out the linkage among the virulence gene profiles, the PMQR genes and the β-lactamases as the limited number of isolates.

MLST investigation showed that ST131 (*n* = 10) and ST10 (*n* = 9) accounted for 47.5% of tested isolates, all ST131 and ST10 isolates were associated with the *bla*_CTX−M−15_ enzyme. ST131 clone has high virulence potential all over the world and represents a major public health problem, and it has emerged and disseminated in *E. coli* from dogs in different continents (Ewers et al., 2010; Platell et al., 2011; Harada et al., 2012; Dahmen et al., 2013; Liu et al., 2016). Other STs, such as ST10, ST38, ST69, and ST167 identified in this study were also reported in human and dogs according to the present data from the MLST database, and ST38 clone can play an important role in the worldwide distribution of CTX-M-producing *E. coli* (Pitout, 2012). PFGE is considered to be a highly discriminative subtyping method in epidemiological investigation, our results suggested that some ST131 isolates and ST10 isolates were closely related, and displayed related restriction patterns, respectively. We should pay attention to these isolates in the future study. However, we cannot find the relationship between the virulence gene profiles and the prevalence of β-lactamases and the STs as the relatively small sample size (40 isolates). Alarmingly, one ST648 clone of phylogenetic group D carried simultaneously *bla*_TEM−1_, *bla*_TSHV−12_, *bla*_CTX−M−15_, *bla*_CTX−M−9_, *bla*_CTX−M−14_, *bla*_OXA−48_, *qnrB, qnrD qnrS, qepA*, and *aac(6*′*)-1b-cr*. Two recent studies in Europe suggested that ST648 clone may represent a novel genotype that combines MDR phenotype, extraintestinal virulence and zoonotic potential in companion animals (Huber et al., 2013; Ewers et al., 2014). To our knowledge, this is the first report of co-existence of 11 β-lactamase and PMQR genes in one *E. coli* isolate, which will obviously improve the resistance to cephalosporins, β-lactamase inhibitors and fluoroquinolones. It is an extremely worrisome sign of development of untreatable infections as the use of β-lactams for the treatment of infections caused by *E. coli* has been and will continue to be the main line of defense against these bacterial agents (Silva-Sanchez et al., 2013).

## Conclusion

In summary, all investigated ESC-R *E. coli* isolates from dogs in Shaanxi province expressed MDR phenotype, exhibited high resistance rate to extended-spectrum cephalosporins and fluoroquinolones, showed high prevalence of β-lactamases and PMQR genes, and all PMQR genes were detected in co-existence with at least one β-lantamase gene. The clonal group ST131, *bla*_CTX−M−15_ gene and *aac(6*′*)-Ib-cr* gene were the dominant ST, β-lactamase gene and PMQR gene among the tested isolates, respectively. This is also the first description of the co-existence of *bla*_TEM−1_, *bla*_SHV−12_, *bla*_CTX−M−15_, *bla*_CTX−M−9_, *bla*_CTX−M−14_, *bla*_OXA−48_, *qnrB, qnrD qnrS, qepA*, and *aac(6*′*)-Ib-cr* genes in one *E. coli* isolate. High prevalence and combination of β-lactamases and PMQR genes is an extremely worrisome sign of treatment of the infections.

## Author contributions

XL conceived and designed the experiments. HL designed the experiment and drafted the manuscript. XL, HL and CH performed the experiments. XL, YL analyzed and explained the data for the work. All authors critically revised and approved the final manuscript.

### Conflict of interest statement

The authors declare that the research was conducted in the absence of any commercial or financial relationships that could be construed as a potential conflict of interest.

## Supplementary material

Figure S1 ***Xba*I-generated pulse-field gel electrophoresis (PFGE) patterns of six ST131 (Y4, Y8, X9, Y11, Y17, and W13 from lane 2–7) and six ST10 (Y13, Y15, B3, W15, W6, and B1 from lane 9–14) isolates**. M, *Salmonella* marker (lane 1, 8, 15).

Table S1 **The Oligonucleotide primers used in this study**.
