# Supplementary figures and images for: High Prevalence of β-lactamase and Plasmid-Mediated Quinolone Resistance Genes in Extended-Spectrum Cephalosporin-Resistant Escherichia coli from Dogs in Shaanxi, China
Source: Front Microbiol. 2016 Nov 16;7:1843. doi: 10.3389/fmicb.2016.01843 (PMC5111280; doi:10.3389/fmicb.2016.01843)

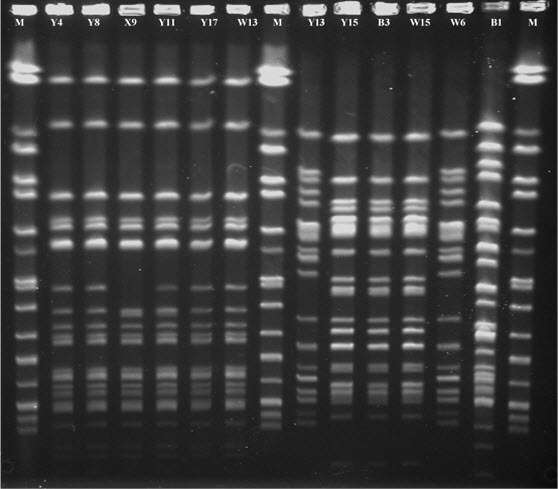

Supplement: Figure S1 — XbaI-generated pulse-field gel electrophoresis (PFGE) patterns of six ST131 (Y4, Y8, X9, Y11, Y17, and W13 from lane 2–7) and six ST10 (Y13, Y15, B3, W15, W6, and B1 from lane 9–14) isolates. M, Salmonella marker (lane 1, 8, 15). [file Image1.JPEG]
