# Supplementary material for: High Prevalence of β-lactamase and Plasmid-Mediated Quinolone Resistance Genes in Extended-Spectrum Cephalosporin-Resistant Escherichia coli from Dogs in Shaanxi, China
Source: Front Microbiol. 2016 Nov 16;7:1843. doi: 10.3389/fmicb.2016.01843 (PMC5111280; doi:10.3389/fmicb.2016.01843)
Supplement: Table S1 — The Oligonucleotide primers used in this study. [file Table1.DOCX]

**Table S1** The Oligonucleotide primers used in this study

| **Target gene** | **Primer sequence (5’-3’)** | **Fragment size (bp)** | **Reference** |
| --- | --- | --- | --- |
| *fimH* | TGCAGAACGGATAAGCCGTGG/ GCAGTCACCTGCCCTCCGGTA | 508 | ([Johnson and Stell, 2000](#_ENREF_9)) |
| *papA* | ATGGCAGTGGTGTCTTTTGGTG/ CGTCCCACCATACGTGCTCTTC | 717 | ([Johnson and Stell, 2000](#_ENREF_9)) |
| *papC* | GTGGCAGTATGAGTAATGACCGTTA/ ATATCCTTTCTGCAGGGATGCAATA | 205 | ([Johnson and Stell, 2000](#_ENREF_9)) |
| *papG* I | TCGTGCTCAGGTCCGGAATTT/ TCCAGAAATAGCTCATGTAACCCG | 479 | ([Mitsumori et al., 1998](#_ENREF_12)) |
| *papG* II | GGGATGAGCGGGCCTTTGAT/ CGGGCCCCCAAGTAACTCG | 190 | ([Johnson and Brown, 1996](#_ENREF_6)) |
| *papG* III | GGCCTGCAATGGATTTACCTGG/ CCACCAAATGACCATGCCAGAC | 258 | ([Johnson and Brown, 1996](#_ENREF_6)) |
| *sfa/focDE* | CTCCGGAGAACTGGGTGCATCTTAC/CGGAGGAGTAATTACAAACCTGGCA | 410 | ([Lebouguenec et al., 1992](#_ENREF_10)) |
| *afa/draBC* | GGCAGAGGGCCGGCAACAGGC/CCCGTAACGCGCCAGCATCTC | 559 | ([Johnson and Brown, 1996](#_ENREF_6)) |
| *hlyA* | AACAAGGATAAGCACTGTTCTGGCT/ACCATATAAGCGGTCATTCCCGTCA | 1,177 | ([Yamamoto et al., 1995](#_ENREF_16)) |
| *cnfI* | ATCTTATACTGGATGGGATCATCTTGG/ GCAGAACGACGTTCTTCATAAGTAT | 974 | ([Yamamoto et al., 1995](#_ENREF_16)) |
| *kpsM* II | GCGCATTTGCTGATACTGTTG/ CATCCAGACGATAAGCATGAGCA | 272 | ([Johnson and Stell, 2000](#_ENREF_9)) |
| *traT* | GGTGTGGTGCGATGAGCACAG/ CACGGTTCAGCCATCCCTGAG | 290 | ([Johnson and Stell, 2000](#_ENREF_9)) |
| *fyuA* | TGATTAACCCCGCGACGGGAA/ CGCAGTAGGCACGATGTTGTA | 880 | ([Johnson and Stell, 2000](#_ENREF_9)) |
| *iroN* | AAGTCAAAGCAGGGGTTGCCCG/ GACGCCGACATTAAGACGCAG | 667 | This study |
| *ireA* | GATGACTCAGCCACGGGTAA/ CCAGGACTCACCTCACGAAT | 254 | This study |
| *iutA* | GGCTGGACATCATGGGAACTGG/ CGTCGGGAACGGGTAGAATCG | 302 | ([Johnson et al., 1998](#_ENREF_7)) |
| *sat* | TACCCTCCACAACAGAGAATG/ TACCCTCCACAACAGAGAATG | 832 | ([Johnson et al., 2000](#_ENREF_8)) |
| *iha* | CTGGCGGAGGCTCTGAGATCA/ TCCTTAAGCTCCCGCGGCTGA | 827 | ([Johnson et al., 2000](#_ENREF_8)) |
| *malX* | GGACATCCTGTTACAGCGCGCA/ TCGCCACCAATCACAGCCGAAC | 930 | ([Johnson and Stell, 2000](#_ENREF_9)) |
| CTX-M | TTTGCGATGTGCAGTACCAGTAA/ CGATATCGTTGGTGGTGCCATA | 544 | ([Edelstein et al., 2003](#_ENREF_3)) |
| CTX-M-1 | CCCATGGTTAAAAAATCACTG/ CCGTTTCCGCTATTACAAAC | 499 | ([Pitout et al., 2004](#_ENREF_14)) |
| CTX-M-15 | AGAATAAGGAATCCCATGGTT/ ACCGTCGGTGACGATTTTAG | 875 | ([Mendonca et al., 2007](#_ENREF_11)) |
| CTX-M-9 | GTGACAAAGAGAGTGCAACGG/ ATGATTCTCGCCGCTGAAGCC | 857 | ([Costa et al., 2006](#_ENREF_2)) |
| CTX-M-14 | GCTGGAGAAAAGCAGCGGAG/ GTAAGCTGACGCAACGTCTG | 470 | ([Pitout et al., 2004](#_ENREF_14)) |
| CTX-M-123 | AATCACTGCGCCAGTTCA/ TTGTTCATGGCGGTATTGT | 780 | ([He et al., 2013](#_ENREF_5)) |
| CTX-M-64 | TACTTCACCCAGCCTCAA/ CCTTACCCAGACAGAGTGC | 1,900 | ([Gu et al., 2015](#_ENREF_4)) |
| TEM | TTCTTGAAGACGAAAGGGC/ ACGCTCAGTGGAACGAAAAC | 1,150 | ([Brinas et al., 2002](#_ENREF_1)) |
| SHV | CACTCAAGGATGTATTGTG/ TTAGCGTTGCCAGTGCTCG | 850 | ([Brinas et al., 2002](#_ENREF_1)) |
| CMY-2 | ATGATGAAAAAATCGTTATGC/ TTGCAGCTTTTCAAGAATGCGC | 1143 | ([Yan et al., 2004](#_ENREF_17)) |
| DHA-1 | AACTTTCACAGGTGTGCTGGGT/ CCGTACGCATACTGGCTTTGC | 405 | ([Perez-Perez and Hanson, 2002](#_ENREF_13)) |
| FOX-1-5b | AACATGGGGTATCAGGGAGATG/ CAAAGCGCGTAACCGGATTGG | 190 | ([Perez-Perez and Hanson, 2002](#_ENREF_13)) |
| ACC | AACAGCCTCAGCAGCCGGTTA/ TTCGCCGCAATCATCCCTAGC | 346 | ([Perez-Perez and Hanson, 2002](#_ENREF_13)) |
| MIR-1T ACT-1 | TCGGTAAAGCCGATGTTGCGG/ CTTCCACTGCGGCTGCCAGTT | 302 | ([Perez-Perez and Hanson, 2002](#_ENREF_13)) |
| KPC | CGTCTAGTTCTGCTGTCTTG/ CTTGTCATCCTTGTTAGGCG | 798 | ([Poirel et al., 2011](#_ENREF_15)) |
| NDM-1 | GGTTTGGCGATCTGGTTTTC/ CGGAATGGCTCATCACGATC | 699 | ([Poirel et al., 2011](#_ENREF_15)) |
| OXA-48 | TTCAAGCCAAAGGCACGATAG / TCCGAGTTGACTGCCGGGTTG | 702 | ([Brinas et al., 2002](#_ENREF_1)) |

**References**

Brinas, L., Zarazaga, M., Saenz, Y., Ruiz-Larrea, F., Torres, C., 2002. Beta-lactamases in ampicillin-resistant Escherichia coli isolates from foods, humans, and healthy animals. Antimicrobial agents and chemotherapy 46, 3156-3163.

Costa, D., Poeta, P., Saenz, Y., Vinue, L., Rojo-Bezares, B., Jouini, A., Zarazaga, M., Rodrigues, J., Torres, C., 2006. Detection of *Escherichia coli* harbouring extended-spectrum beta-lactamases of the CTX-M, TEM and SHV classes in faecal samples of wild animals in Portugal. The Journal of antimicrobial chemotherapy 58, 1311-1312.

Edelstein, M., Pimkin, M., Palagin, I., Edelstein, I., Stratchounski, L., 2003. Prevalence and molecular epidemiology of CTX-M extended-spectrum beta-lactamase-producing *Escherichia coli* and *Klebsiella pneumoniae* in Russian hospitals. Antimicrobial agents and chemotherapy 47, 3724-3732.

Gu, D.X., Yu, T., Wang, Y., Zhang, R., 2015. Detection of CTX-M-64 in *Escherichia coli* isolates from human patients in China. Antimicrobial agents and chemotherapy 59, 1371-1372.

He, D., Partridge, S.R., Shen, J., Zeng, Z., Liu, L., Rao, L., Lv, L., Liu, J.H., 2013. CTX-M-123, a novel hybrid of the CTX-M-1 and CTX-M-9 Group beta-lactamases recovered from *Escherichia coli* isolates in China. Antimicrobial agents and chemotherapy 57, 4068-4071.

Johnson, J.R., Brown, J.J., 1996. A novel multiply primed polymerase chain reaction assay for identification of variant papG genes encoding the Gal(alpha 1-4)Gal-binding PapG adhesins of *Escherichia coli*. The Journal of infectious diseases 173, 920-926.

Johnson, J.R., Brown, J.J., Carlino, U.B., Russo, T.A., 1998. Colonization with and acquisition of uropathogenic *Escherichia coli* as revealed by polymerase chain reaction-based detection. Journal of Infectious Diseases 177, 1120-1124.

Johnson, J.R., Russo, T.A., Tarr, P.I., Carlino, U., Bilge, S.S., Vary, J.C., Jr., Stell, A.L., 2000. Molecular epidemiological and phylogenetic associations of two novel putative virulence genes, iha and iroN(*E. coli*), among *Escherichia coli* isolates from patients with urosepsis. Infection and immunity 68, 3040-3047.

Johnson, J.R., Stell, A.L., 2000. Extended virulence genotypes of *Escherichia coli* strains from patients with urosepsis in relation to phylogeny and host compromise. The Journal of infectious diseases 181, 261-272.

Lebouguenec, C., Archambaud, M., Labigne, A., 1992. Rapid and Specific Detection of the Pap, Afa, and Sfa Adhesin-Encoding Operons in Uropathogenic *Escherichia-Coli* Strains by Polymerase Chain-Reaction. Journal of clinical microbiology 30, 1189-1193.

Mendonca, N., Leitao, J., Manageiro, V., Ferreira, E., Canica, M., 2007. Spread of extended-spectrum beta-lactamase CTX-M-producing *escherichia coli* clinical isolates in community and nosocomial environments in Portugal. Antimicrobial agents and chemotherapy 51, 1946-1955.

Mitsumori, K., Terai, A., Yamamoto, S., Yoshida, O., 1998. Identification of S, F1C and three PapG fimbrial adhesins in uropathogenic *Escherichia coli* by polymerase chain reaction. FEMS immunology and medical microbiology 21, 261-268.

Perez-Perez, F.J., Hanson, N.D., 2002. Detection of plasmid-mediated AmpC beta-lactamase genes in clinical isolates by using multiplex PCR. Journal of clinical microbiology 40, 2153-2162.

Pitout, J.D., Hossain, A., Hanson, N.D., 2004. Phenotypic and molecular detection of CTX-M-beta-lactamases produced by Escherichia coli and Klebsiella spp. Journal of clinical microbiology 42, 5715-5721.

Poirel, L., Walsh, T.R., Cuvillier, V., Nordmann, P., 2011. Multiplex PCR for detection of acquired carbapenemase genes. Diagnostic microbiology and infectious disease 70, 119-123.

Yamamoto, S., Terai, A., Yuri, K., Kurazono, H., Takeda, Y., Yoshida, O., 1995. Detection of Urovirulence Factors in *Escherichia-Coli* by Multiplex Polymerase Chain-Reaction. FEMS immunology and medical microbiology 12, 85-90.

Yan, J.J., Hong, C.Y., Ko, W.C., Chen, Y.J., Tsai, S.H., Chuang, C.L., Wu, J.J., 2004. Dissemination of blaCMY-2 among *Escherichia coli* isolates from food animals, retail ground meats, and humans in southern Taiwan. Antimicrobial agents and chemotherapy 48, 1353-1356.
